# The College of Nursing and the State Recognition of Nurses

**Published:** 1919-04-05

**Authors:** 


### The Hospital

The Workers' Newspaper of Administrative Medicine and Institutional Life, Administration, National Insurance and Health.

No. 1713, Vol. LXVI. SATURDAY, APRIL 5, 1919. PRICE TWOPENCE

### THE COLLEGE OF NURSING AND THE STATE RECOGNITION OF NURSES.

The College of Nursing has for the past three years done all in its power to bring the need for State recognition of the nursing profession prominently before the public and the nurses themselves, and it is very glad that its efforts have led to the House giving a second reading to the State Registration Bill without a division. In accordance with its policy the College supported the Bill, feeling that any differences as to procedure could very well be settled in Committee. Colonel Raw and Mr. Lyle, with other friends of the College in the House, will no doubt be on the Standing Committee and will see that the amendments to the Bill which the College thinks vital are duly brought forward and considered.

It is quite clear that the Bill can only go through if it has the approval of the Government, and if it has that approval it may be taken for granted that the Bill will be in such a form that the College of Nursing can also support it. In this connection the remark of the Parliamentary Secretary of the Local Government Board (Major Astor) in the House on March 28 should be noted, viz.: "Of course the attitude which the Government will take up when the Bill comes down here must of necessity depend upon its shape then." The College hopes that the Bill will come down from the Standing Committee in such a shape that the Government will be able to adopt it as a Government measure. It is obviously right, in view of the establishment of a Ministry of Health, that a Bill for the State Registration of Nurses should be in the hands of that Ministry.

The need for the College of Nursing will be found even greater after the Bill is passed than now, for an Act of this kind will be of little value to the nurses unless there is a strong association of the nurses themselves ready and able to take advantage of the powers obtained under the Act. The *Times*, in its leading article on Saturday, March 29, points out very rightly that the great thing is to get the opinion of the individual nurses. The College, with its membership of 12,600 nurses —a membership which is growing every day—and through its organisation of local centres and nurse clubs throughout the whole country, is in a position to speak for the nurses themselves, and to give them a clear and definite voice in the shaping of the policy of their own profession.

Moreover, as the nursing profession will now, it is hoped, receive the recognition of the State, problems of education become even more important than they were before, and it is to deal with these very problems that the College has been established. Altogether the future of the College of Nursing looks very bright, and we learn that personally Sir Arthur Stanley feels confident that the nurses who have helped to build up the College will have every reason to feel satisfied with their work, and that the College will be one of the permanent, and certainly one of the most important, Memorials of the Great War. We agree with Sir Arthur in holding these views, and believe that every member on the Register of the College will work with a will, and continuously to recruit members to join the College in ever-increasing numbers.

The debate in the House of Commons on the Second Reading was well sustained, and revealed a general wish to create a nursing profession on a State basis which should do justice to the trained nurse, protect her uniform, secure her a minimum but adequate remuneration, and put a permanent stop to sweating and over-work everywhere throughout the country. The principle of nurse registration was accepted unanimously, but the fate of the Bill submitted rests upon the thorough remodelling of the Central Committee's Bill in Committee, and its being made acceptable to the Government and consequently to the College if it is to pass. The mover, Major Barnett, in a very moderate statement appealed "to every Member of Parliament who is in favour of the principle to vote for the Second Reading and to raise any objections he may have in Committee." Colonel Wedgwood clearly set forth the fatal objections to the Bill as it passed the Second Reading, and rightly insisted that it only put one side before the House of Commons. Members must remember that at the end of three years they would have to approach the nursing profession through a certain channel. That channel, as we all know by the action of those responsible for this Bill in the past, is the main cause of the present disagreement, and such policy and methods must be radically changed or the Bill cannot pass. This was made perfectly clear throughout the debate and by the speech of Major Astor, the Parliamentary Secretary to the Local Government, who represented the Government.

